# Redefining the Role of Splenectomy in Scirrhous Gastric Cancer: Toward a Selective Multimodal Strategy

**DOI:** 10.1002/ags3.70254

**Published:** 2026-07-25

**Authors:** Tsutomu Hayashi, Takaki Yoshikawa

**Affiliations:** ^1^ Department of Gastric Surgery National Cancer Center Hospital Tokyo Japan; ^2^ Department of Gastrointestinal Surgery Yokohama City University Yokohama Japan

**Keywords:** minimally invasive surgery, neoadjuvant therapy, scirrhous gastric cancer, splenectomy, splenic hilar lymph node

## Abstract

Scirrhous gastric cancer, characterized by diffuse infiltration and a high propensity for peritoneal dissemination, remains one of the most aggressive subtypes of gastric cancer. Despite a relatively high incidence of splenic hilar lymph node (No. 10) metastasis, the oncologic benefit of splenectomy remains controversial, while its association with increased postoperative morbidity is well established. Recent advances in systemic therapy, including immune checkpoint inhibitors and molecular‐targeted agents, have shifted the treatment paradigm toward improved systemic disease control. In parallel, minimally invasive surgical approaches, particularly spleen‐preserving splenic hilar lymph node dissection, have emerged as feasible alternatives to conventional splenectomy. However, in scirrhous gastric cancer, disease‐specific features such as diffuse infiltration, marked fibrosis, and difficulty in intraoperative assessment continue to pose significant challenges. Furthermore, the optimal extent of lymphadenectomy, especially following neoadjuvant therapy, remains undefined. Taken together, splenectomy should no longer be regarded as a routine component of surgical management for scirrhous gastric cancer, but rather as a selective procedure tailored to individual tumor characteristics and treatment context. Future studies integrating systemic therapy, surgical safety, and oncologic adequacy are required to establish evidence‐based strategies for this challenging disease.

## Abstract 2

Scirrhous gastric cancer is characterized by diffuse infiltration and a high propensity for peritoneal dissemination, which may limit the impact of locoregional control strategies alone. Although splenic hilar lymph node metastasis occurs relatively frequently, the oncologic benefit of routine splenectomy remains uncertain, whereas increased postoperative morbidity is well established. In the modern era of multimodal treatment, splenectomy should be reconsidered as a selective procedure based on individual tumor characteristics and treatment context.

## 1 Introduction

Scirrhous gastric cancer, corresponding to Borrmann type 4 disease, is characterized by diffuse infiltrative growth and represents one of the most aggressive subtypes of gastric cancer. Despite advances in surgical techniques and perioperative management, long‐term outcomes remain poor. Reported 5‐year survival rates range from 6.3% to 38%, consistently lower than those observed in other macroscopic types of gastric cancer [1, 2, 3].

The most critical clinical challenge in scirrhous gastric cancer is its high rate of peritoneal recurrence. Previous studies have reported that peritoneal recurrence accounts for up to 76.8% of recurrences following curative resection [2]. Similarly, in the JCOG0501 randomized trial, peritoneal dissemination was observed in 76.4% of patients in the neoadjuvant chemotherapy arm and 80.2% in the surgery‐first arm, indicating that peritoneal metastasis is the dominant mode of failure in this disease [4].

These findings suggest that scirrhous gastric cancer behaves more like a systemic disease characterized by early peritoneal dissemination rather than a purely locoregional malignancy. Consequently, local control strategies alone, including extended lymphadenectomy, may have limited impact on long‐term outcomes. In this context, the survival benefit associated with prophylactic lymph node dissection aimed at preventing nodal recurrence may differ from that observed in conventional advanced gastric cancer.

Evidence from Japanese randomized trials in advanced gastric cancer provides a useful framework for understanding the differences in clinical behavior between conventional advanced gastric cancer and scirrhous gastric cancer. JCOG0110 [5, 6] evaluated the role of splenectomy in proximal gastric cancer, whereas JCOG1001 investigated the value of bursectomy in patients with locally advanced gastric cancer. In contrast, JCOG0501 specifically enrolled patients with type 4 and large type 3 gastric cancer and therefore serves as the principal randomized evidence base for scirrhous gastric cancer. As summarized in Table 1, recurrence patterns and long‐term outcomes differ between conventional advanced gastric cancer and scirrhous gastric cancer. In JCOG1001 [6], hematogenous and peritoneal recurrence occurred at similar frequencies, whereas peritoneal dissemination accounted for nearly 80% of recurrences in JCOG0501. Furthermore, survival remained inferior in patients with scirrhous gastric cancer despite neoadjuvant chemotherapy. These findings suggest that prognosis in scirrhous gastric cancer is largely determined by peritoneal disease rather than locoregional failure alone, potentially reducing the relative contribution of extensive lymphadenectomy to long‐term outcomes. This distinction raises important questions regarding the applicability of evidence derived from conventional gastric cancer to scirrhous gastric cancer and highlights the need for disease‐specific evaluation of the role of splenectomy in this setting.

**TABLE 1:** Key characteristics and recurrence patterns of representative Japanese randomized trials relevant to the role of splenectomy.

	Non‐scirrhous		Scirrhous
	JCOG0110	JCOG1001	JCOG0501
Disease	Proximal GC without greater curvature involvement	Advanced GC	Type 4/large Type 3 GC
Clinical stage	cT2‐4, any N	cT3‐4a, any N, CY0 or 1	cT2‐4, any N, CY0 or 1
Trial intervention	Splenectomy vs. spleen preservation	Bursectomy vs. non‐bursectomy	NAC vs. upfront surgery
Overall survival	5‐year OS: 75%–76%	5‐year OS: 76%–77%	3‐year OS: 60%–62%
Initial recurrence pattern^a^	Not described	Peritoneal: 41% Lymph nodes: 28% Hematogenous: 45%	Peritoneal: 78% Lymph nodes: 14% Hematogenous: 14%

^a^ Patients with multiple recurrence sites at initial recurrence were included in each category; therefore, percentages may exceed 100%.

However, the optimal extent of lymphadenectomy, particularly the role of splenectomy for splenic hilar lymph node dissection, remains controversial. Major randomized trials evaluating extended lymphadenectomy have largely excluded patients with scirrhous gastric cancer, resulting in a lack of high‐level evidence specific to this subgroup. Therefore, the clinical value of splenectomy in scirrhous gastric cancer remains undefined.

In this review, the term “scirrhous gastric cancer” is used in a broad clinical context to include Borrmann type 4 tumors, linitis plastica, and other diffuse infiltrative advanced gastric cancers, including large type 3 lesions with scirrhous characteristics. Within this framework, we review the available evidence regarding recurrence patterns, lymph node metastasis, the therapeutic value of splenic hilar lymph node dissection, and evolving surgical strategies in order to evaluate the current role of splenectomy in scirrhous gastric cancer.

## 2 Biological Characteristics of Scirrhous Gastric Cancer

Scirrhous gastric cancer, corresponding to diffuse‐type gastric cancer (DGC) in the Lauren classification, is characterized by a unique biological behavior distinct from intestinal‐type tumors. Unlike gland‐forming cancers, DGC is composed of poorly cohesive cells that infiltrate diffusely within the gastric wall, often without forming a discrete mass. This infiltrative growth pattern is closely associated with alterations in cell–cell adhesion, most notably loss of E‐cadherin through CDH1 inactivation [7, 8]. Disruption of E‐cadherin‐mediated adherens junctions reduces intercellular cohesion and promotes cellular motility, thereby facilitating diffuse tumor infiltration.

Beyond the loss of cell adhesion, DGC exhibits a prominent desmoplastic reaction, characterized by extensive fibrosis and a stromal‐rich tumor microenvironment. TGF‐β signaling drives the accumulation of activated fibroblasts and extracellular matrix, contributing to the rigid gastric morphology and promoting tumor progression through cancer–stroma crosstalk [9]. Increased stromal gene expression signatures have been associated with more aggressive tumor behavior and poorer clinical outcomes in DGC [9].

At the molecular level, DGC is frequently associated with genetic alterations involving CDH1, CTNNA1, and RHOA, among others, which collectively contribute to impaired cell adhesion, cytoskeletal remodeling, and enhanced invasive potential [8, 9]. In particular, loss of E‐cadherin function disrupts epithelial integrity and activates multiple downstream signaling pathways that regulate actomyosin dynamics and cellular migration [7]. These alterations are thought to underlie the transition from early intramucosal lesions composed of signet‐ring cells to highly invasive tumors capable of widespread dissemination.

Taken together, these biological features—including impaired cell adhesion, stromal‐rich tumor microenvironment, and enhanced invasive capacity—create a tumor phenotype that is predisposed to diffuse infiltration and early dissemination beyond the primary lesion. These mechanisms may partly explain why the impact of local control strategies, including extended lymphadenectomy, could differ from that observed in conventional gastric cancer.

## 3 Recurrence Pattern and Implications for Surgical Strategy

The predominance of peritoneal dissemination in scirrhous gastric cancer has important implications for surgical strategy. Unlike intestinal‐type gastric cancer, in which locoregional control plays a central role in improving outcomes, scirrhous gastric cancer is driven primarily by early systemic peritoneal spread, suggesting that tumor dissemination beyond regional lymphatic boundaries occurs at an early stage. Consequently, optimal locoregional resection alone may be insufficient to alter the natural history of the disease. This raises critical questions regarding the oncologic value of extended lymphadenectomy, particularly prophylactic dissection of the splenic hilar lymph nodes.

Therefore, in the context of scirrhous gastric cancer, the potential survival benefit of aggressive nodal dissection should be interpreted with caution. Rather than focusing solely on the extent of lymphadenectomy, a more balanced approach that prioritizes systemic disease control and treatment tolerability may be warranted, providing the rationale for further evaluation of the role of splenectomy in this disease.

## 4 Lymph Node Metastasis and Oncologic Value of No. 10 Dissection

Despite its diffuse infiltrative growth pattern, scirrhous gastric cancer is frequently associated with extensive lymph node metastasis. Previous studies have reported that lymph node involvement is present in more than 70% of patients, with metastases distributed across multiple nodal stations [2, 3, 10]. High metastatic rates have been observed not only in perigastric lymph nodes, such as No. 1, No. 3, and No. 4d, but also in more distant regional stations, including No. 7, No. 10, and No. 11 nodes, indicating widespread lymphatic dissemination. Furthermore, No. 10‐positive patients often exhibit advanced nodal disease; Kunimoto et al. [2] reported that 87.5% of patients with splenic hilar metastasis had pN3 disease. These findings provide an important background for evaluating the role of No. 10 lymph node dissection and the appropriate extent of lymphadenectomy in scirrhous gastric cancer.

Among regional nodal stations, the splenic hilar lymph nodes (No. 10) have attracted particular attention because metastasis to this station is not uncommon in scirrhous gastric cancer. As summarized in Table 2, the reported incidence of No. 10 lymph node metastasis in type 4 gastric cancer ranges from 14.2% to 28.6% in most series [2, 3, 10, 11, 12, 13, 14, 15, 16], indicating that splenic hilar involvement occurs relatively frequently in this disease. These rates are generally comparable to, or higher than, those reported in conventional proximal gastric cancer. Furthermore, studies directly comparing type 4 and non‐type 4 tumors have reported higher rates of No. 10 metastasis in type 4 gastric cancer [11, 13], although this finding has not been consistently observed across all studies. Collectively, these findings suggest that the splenic hilar lymph nodes represent a clinically relevant metastatic site in scirrhous gastric cancer and provide an oncologic rationale for considering No. 10 lymph node dissection.

**TABLE 2:** Incidence of splenic hilar lymph node (No. 10) metastasis.

Year	Author	Population	Incidence of No. 10 lymph node metastasis, % (*n*/*N*)		Citations
			Type 4	Non‐type 4	
2011	Kosuga	Advanced proximal GC	26.4% (19/72)	5.29% (11/208)	[11]
2016	Watanabe	Advanced proximal GC	14.2% (8/56)	17.1% (13/76)	[12]
2017	Son	Advanced GC undergoing TG	24.5% (35/143)	11.3% (52/459)	[13]
2018	Maezawa	Proximal GC with greater‐curvature invasion	13.4% (11/82)		[14]
2019	Yura	Proximal GC with greater‐curvature invasion	15.1% (32/212)		[15]
2020	Hayashi	Type 4 GC	15.3% (21/137)	—	[3]
2020	Kano	Proximal GC with greater‐curvature invasion	26.0% (13/50)	31.7% (19/60)	[16]
2021	Kunimoto	Type 4 GC	28.6% (16/56)	—	[2]
2025	Fujisaki	Type 4 (antral type)	0% (0/19)	—	[10]
2025	Fujisaki	Type 4 (body‐type)	21.7% (35/161)	—	[10]

*Note:* Studies differed substantially in patient selection and disease extent. Some studies directly compared type 4 and non‐type 4 gastric cancer, whereas others evaluated only type 4 gastric cancer or conventional proximal gastric cancer. Therefore, direct comparison across studies should be interpreted with caution.

Abbreviations: GC, gastric cancer; TG, total gastrectomy.

However, the clinical significance of lymph node dissection should not be assessed based on metastatic incidence alone. The therapeutic value index (TVI), which integrates the incidence of nodal metastasis with survival outcomes of patients with positive nodes, provides a more comprehensive measure of the survival benefit associated with lymph node dissection.

As summarized in Table 3, studies reporting similar incidences of No. 10 metastasis have not necessarily demonstrated similar therapeutic value of No. 10 dissection. For example, Kunimoto et al. [2] reported a No. 10 metastatic rate of 28.6%, but the 5‐year survival rate among patients with No. 10 metastasis was only 6.7%, resulting in a TVI of 1.92 and ranking 15th among 16 evaluated nodal stations. In contrast, Hayashi et al. [3] reported a lower metastatic rate of 15.3%, but the corresponding 5‐year survival rate was 33.3%, resulting in a TVI of 5.09. Similarly, Kosuga et al. [11] reported a TVI of 12.9, which was higher than that reported in other type 4 cohorts. These findings suggest that differences in TVI are influenced not only by the incidence of No. 10 metastasis but also by the prognosis of patients with metastatic involvement of this station.

**TABLE 3:** Therapeutic index (TI) of splenic hilar lymph node (No. 10).

Year	Author		Therapeutic index of No. 10 nodes dissection		Citations
			Type 4 (priority)	Non‐type 4 (priority)	
2011	Kosuga	Advanced proximal GC	12.9 (high)	2.98 (moderate low)	[11]
2016	Watanabe	Advanced proximal GC	7.1 (moderate high)	4.0 (moderate low)	[12]
2017	Son	Advanced GC undergoing TG	3.5 (moderate high)		[13]
2018	Maezawa	Proximal GC with greater‐curvature invasion	4.02 (9/15)		[14]
2019	Yura	Proximal GC with greater‐curvature invasion	7.1 (8/14)		[15]
2020	Hayashi	Type 4 GC	5.09 (6/15)	—	[3]
2020	Kano	Proximal GC with greater‐curvature invasion	13.7 (9/15)	15.0 (2/15)	[16]
2021	Kunimoto	Type 4 GC	1.92 (15/16)	—	[2]
2025	Fujisaki	Type 4 (antral‐type)	0		[10]
2025	Fujisaki	Type 4 (body‐type)	7.75 (4/14)		[10]

*Note:* The therapeutic index (TI) was calculated as the incidence of No. 10 metastasis multiplied by the 5‐year survival rate of patients with metastasis at this station. Because TI is primarily intended to evaluate the relative contribution of individual nodal stations within the same cohort, direct comparison of absolute TI values across different studies should be interpreted with caution. Therefore, the relative ranking of No. 10 among evaluated nodal stations within each study is shown as an indicator of its relative therapeutic value. Priority reflects the relative position of No. 10 among evaluated nodal stations within the same study, expressed either as rank order when reported or as a qualitative classification based on the reported TI.

Abbreviations: GC, gastric cancer; TG, total gastrectomy.

One possible explanation is that No. 10 metastasis occurs in different clinical contexts across study populations. In some cohorts, splenic hilar nodal metastasis may represent a potentially controllable regional disease, whereas in others it may occur as part of extensive nodal dissemination and systemic disease progression. Notably, Kunimoto et al. [2] reported that 87.5% of patients with No. 10 metastasis had pN3 disease, and R1 resection was observed in approximately one‐third of No. 10‐positive patients. These findings suggest that splenic hilar nodal metastasis frequently occurred in patients with extensive disease at the time of diagnosis. Under such circumstances, No. 10 metastasis may be more indicative of advanced systemic disease than a target for locoregional control alone, which could partly explain the low TVI observed in this cohort. The marked variation in TVI across studies suggests that the oncologic value of No. 10 dissection is not uniform across all patients with scirrhous gastric cancer and may depend on individual clinicopathological characteristics.

A comparison with conventional proximal gastric cancer provides additional insight into the interpretation of TVI. In non‐type 4 gastric cancer, the reported therapeutic value of No. 10 dissection varies according to tumor characteristics. However, studies focusing on proximal tumors with greater curvature involvement [14, 15, 16] have consistently reported intermediate rankings of No. 10 among D2 nodal stations, supporting the clinical relevance of splenic hilar dissection in this setting. In contrast, substantially greater variation has been observed in scirrhous gastric cancer. While some studies have reported moderate‐to‐high relative rankings of No. 10, others have demonstrated limited therapeutic value despite relatively high metastatic rates. These findings suggest that the oncologic value of No. 10 dissection may be more strongly influenced by disease extent and clinicopathological characteristics in scirrhous gastric cancer than in conventional proximal gastric cancer.

Recent evidence further suggests that the oncologic relevance of No. 10 dissection may differ even within type 4 gastric cancer. Fujisaki et al. [10] reported no No. 10 metastasis in antral‐type scirrhous gastric cancer, whereas body‐type tumors demonstrated a metastatic rate of 21.7% and a relatively favorable TVI. Previous studies have also identified greater curvature involvement and extensive serosal invasion as potential risk factors for No. 10 metastasis [12]. Although the available evidence remains limited, these findings support a selective approach to No. 10 dissection based on individual tumor characteristics rather than routine application in all patients with scirrhous gastric cancer.

Taken together, the available evidence suggests that the indication for No. 10 dissection should be interpreted in the context of both metastatic risk and therapeutic value. Disease extent and clinicopathological characteristics should also be taken into account when evaluating its role in scirrhous gastric cancer.

## 5 Evidence on the Harms of Splenectomy

Splenectomy combined with total gastrectomy has traditionally been performed as a standard approach to ensure complete dissection of the splenic hilar lymph nodes (No. 10). However, the invasiveness of this procedure has long been a major concern, particularly with respect to an increased risk of pancreas‐related complications. Previous studies have consistently reported higher rates of postoperative morbidity, including pancreatic fistula and intra‐abdominal abscess, associated with splenectomy.

The JCOG0110 trial, a large‐scale randomized controlled study evaluating the role of splenectomy in proximal advanced gastric cancer, demonstrated that the splenectomy group had a significantly higher incidence of postoperative complications and greater intraoperative blood loss compared with the spleen‐preserving group [17]. Importantly, no significant difference in overall survival was observed, and the non‐inferiority of spleen‐preserving surgery was confirmed. In the study population, prophylactic splenectomy increased surgical morbidity without improving survival. Because patients with scirrhous gastric cancer and tumors involving the greater curvature were excluded, these findings cannot be directly extrapolated to these populations.

Consistent results have been reported in meta‐analyses. In a large meta‐analysis including 4457 patients, splenectomy was associated with a significantly higher rate of postoperative complications (relative risk [RR] 1.68), including pancreatic fistula (RR 2.67), intra‐abdominal abscess (RR 3.16), and anastomotic leakage (RR 1.58) [18]. These findings further support the increased surgical morbidity associated with splenectomy, without demonstrating a clear survival advantage.

Similar trends have been observed in minimally invasive surgery. Comparative studies of laparoscopic total gastrectomy have demonstrated a higher incidence of pancreatic fistula in the splenectomy group compared with spleen‐preserving procedures (15.8% vs. 5%) [19]. In addition, retrospective analyses in real‐world clinical settings have shown significantly greater blood loss (538 vs. 450 mL) and higher postoperative complication rates (30.2% vs. 13.3%) in patients undergoing splenectomy [20]. In particular, pancreas‐related complications were observed in up to 17.4% of patients in the splenectomy group, whereas no such complications were reported in the spleen‐preserving group, suggesting that manipulation around the pancreas during splenectomy contributes substantially to postoperative morbidity.

Beyond surgical morbidity, the impact of splenectomy on host immune function should also be considered. The spleen is the largest lymphoid organ and plays a critical role in both innate and adaptive immunity, including tumor immune surveillance [21]. Splenectomy may impair these immune functions, potentially increasing not only the risk of infectious complications but also the risk of malignancy. Indeed, large cohort studies have reported a significantly increased overall risk of cancer development following splenectomy [22]. However, in the long‐term follow‐up of the JCOG0110 trial, no significant difference in the incidence of infectious events was observed between the splenectomy and spleen‐preserving groups [17]. This suggests that clinically overt immunodeficiency may not be evident in the short term, although subclinical immune alterations may have long‐term consequences for tumor surveillance.

Importantly, patients with tumors involving the greater curvature and those with scirrhous gastric cancer (Borrmann type 4) were excluded from the JCOG0110 trial. Therefore, the results of this study cannot be directly extrapolated to these subgroups, and the validity of omitting splenectomy in scirrhous gastric cancer remains uncertain.

Current clinical guidelines continue to include splenic hilar lymph node dissection as a recommended component of surgical treatment for selected patients with proximal gastric cancer. However, the increased risk of postoperative morbidity associated with splenectomy has been consistently demonstrated. Therefore, in patients with scirrhous gastric cancer, the decision to perform splenectomy should be based on a careful balance between its potential oncologic benefit and the increased risk of postoperative morbidity, rather than uniform application in all patients.

## 6 Evolving Role of Systemic Therapy

Recent advances in perioperative treatment have provided an important context for reconsidering surgical strategies in scirrhous gastric cancer. The JCOG0501 trial demonstrated no additional survival benefit of neoadjuvant chemotherapy with S‐1 plus cisplatin, highlighting the limitations of conventional cytotoxic regimens in this disease [4].

In contrast, the introduction of immune checkpoint inhibitors and molecularly targeted therapies has begun to improve outcomes in gastric cancer. In the MATTERHORN trial, the addition of durvalumab to perioperative FLOT chemotherapy resulted in improved event‐free survival (EFS). Although variability in treatment effect has been suggested in diffuse‐type tumors, which include scirrhous gastric cancer, these findings indicate a potential shift toward more effective systemic control [23]. Furthermore, CLDN18.2‐targeted therapies have emerged as a promising option, particularly in diffuse‐type gastric cancer, where expression of this target is relatively frequent [24, 25].

Against this evolving therapeutic landscape, the role of surgery is evolving. While extended lymphadenectomy was traditionally emphasized for locoregional control, increasing attention is now paid to continuity of multimodal treatment, including timely initiation of postoperative systemic therapy. Splenectomy, which is associated with an increased risk of postoperative complications, may compromise treatment continuity by delaying or limiting the delivery of adjuvant therapy.

Given that peritoneal dissemination is the predominant determinant of prognosis in scirrhous gastric cancer, the relative benefit of more extensive locoregional dissection may be inherently limited. Therefore, in the modern treatment era, it is necessary to reconsider the extent of prophylactic lymphadenectomy, particularly the role of splenectomy, in light of the balance between surgical invasiveness and systemic disease control.

## 7 Minimally Invasive Surgery and Spleen‐Preserving Approaches

Recent advances in laparoscopic and robotic surgery have made spleen‐preserving splenic hilar lymph node dissection (No. 10) an increasingly feasible and clinically relevant option. Traditionally, the splenic hilum has been considered a technically challenging area due to its complex vascular anatomy and anatomical variability, and splenectomy has often been performed to facilitate adequate lymph node dissection. However, improvements in minimally invasive techniques, including magnified visualization and precise instrument control, have enabled safe and effective No. 10 dissection while preserving the spleen.

The feasibility and safety of laparoscopic spleen‐preserving No. 10 dissection have been demonstrated in prospective multicenter studies. In a prospective multicenter trial conducted from Korea [26], an analysis of 242 patients reported a postoperative complication rate of 13.6% and a major complication rate of 3.3%, both within acceptable ranges. The incidence of No. 10 lymph node metastasis was 8.1% in this cohort. Furthermore, comparative studies between spleen‐preserving and splenectomy approaches in laparoscopic surgery [19] have shown comparable numbers of retrieved lymph nodes, while intraoperative blood loss and pancreas‐related complications tend to be lower in the spleen‐preserving group. These findings support the technical feasibility and short‐term safety of spleen‐preserving No. 10 dissection, while its long‐term oncologic equivalence remains to be established.

Robotic‐assisted surgery has further enhanced the technical feasibility of spleen‐preserving No. 10 dissection. With articulated instruments, tremor filtration, and three‐dimensional visualization, robotic systems enable stable and precise manipulation in confined and anatomically complex regions such as the splenic hilum. Several studies [27, 28] have reported that robotic spleen‐preserving splenic hilar dissection can be performed safely, with reduced blood loss and accurate lymph node retrieval. However, the available evidence is based largely on retrospective studies, and robust data on long‐term oncologic outcomes are still lacking.

In the context of scirrhous gastric cancer, the application of minimally invasive surgery has been gradually explored. A comparative study of laparoscopic/robot‐assisted versus open surgery for Borrmann type 4 gastric cancer demonstrated comparable postoperative morbidity and oncologic outcomes, suggesting the feasibility of MIS in selected patients. Additional retrospective analyses have similarly reported comparable survival outcomes between minimally invasive and open approaches [29, 30, 31]. These results suggest that MIS can be incorporated into the management of scirrhous gastric cancer with appropriate patient selection.

However, several disease‐specific challenges must be carefully considered. First, the risk of tumor cell dissemination associated with instrument manipulation has been raised as a potential concern. Given the diffuse infiltrative nature and fragile tumor architecture, excessive grasping or traction of the gastric wall may theoretically promote cancer cell exfoliation and peritoneal dissemination. Second, the marked fibrosis and diffuse thickening of the gastric wall characteristic of scirrhous gastric cancer often make adequate traction and exposure technically difficult. Compared with open surgery, where direct manual handling allows flexible adjustment of tension and exposure, minimally invasive approaches may be limited in achieving a stable operative field, particularly in cases with extensive serosal involvement or whole‐stomach infiltration. Third, the lack of tactile feedback inherent to laparoscopic and robotic surgery may hinder intraoperative assessment of tumor extent and margins. In scirrhous gastric cancer, where tumor boundaries are often indistinct and submucosal spread is extensive, accurate determination of resection lines can be challenging.

In addition to these technical challenges, spleen‐preserving No. 10 dissection itself remains a technically demanding procedure that requires a high level of anatomical understanding and advanced surgical expertise. Even in prospective clinical trials, strict criteria for surgeon experience and technical proficiency have been applied, underscoring the importance of adequate training for safe implementation [26]. Therefore, careful patient selection and consideration of institutional and surgeon experience are essential when adopting this technique.

In this context, a prospective multicenter phase II trial (JCOG1809 investigators, Japan Clinical Oncology Group) has been conducted in Japan to evaluate the feasibility and oncologic validity of spleen‐preserving splenic hilar lymph node dissection [32]. Patient enrollment has been completed, and the results are awaited. This study aims to establish a less invasive alternative to splenectomy while maintaining oncologic adequacy, and its findings are expected to have a significant impact on future surgical practice.

In summary, advances in minimally invasive surgery have enabled spleen‐preserving No. 10 dissection as a technically feasible and less invasive alternative to splenectomy. Nevertheless, its role in scirrhous gastric cancer remains to be established because of disease‐specific technical challenges and the lack of prospective oncologic evidence. Further prospective studies specifically addressing patients with scirrhous gastric cancer are warranted.

## 8 Future Directions

The role of splenectomy in the surgical management of scirrhous gastric cancer warrants reconsideration in the modern treatment era. Although current guidelines recommend D2 lymphadenectomy including splenic hilar lymph node dissection for selected patients with proximal advanced gastric cancer, the applicability of this recommendation to scirrhous gastric cancer remains uncertain.

From an oncologic perspective, the variable therapeutic value of No. 10 dissection reported across studies suggests that the role of splenectomy should be reconsidered. The survival benefit associated with splenectomy remains inconsistent, while its association with increased postoperative morbidity is well established. Taken together, these observations highlight the need to redefine the role of splenectomy in this disease entity.

The emergence of more effective systemic therapies adds further complexity. Neoadjuvant regimens incorporating immune checkpoint inhibitors and molecularly targeted agents are expected to improve systemic disease control, potentially reducing the relative contribution of extensive lymphadenectomy to overall survival. However, the optimal extent of lymph node dissection following neoadjuvant therapy remains largely undefined, particularly in scirrhous gastric cancer, where high‐level evidence is lacking.

In this context, spleen‐preserving splenic hilar lymph node dissection offers a promising alternative, enabling adequate nodal clearance while avoiding splenectomy‐related morbidity and preserving treatment continuity. Ongoing prospective studies, including JCOG1809, are expected to clarify the oncologic validity and clinical applicability of this technique. However, evidence supporting its oncologic efficacy, particularly in patients with scirrhous gastric cancer, remains limited, and further prospective validation is required.

At the same time, the adoption of minimally invasive approaches introduces additional considerations. Although laparoscopic and robotic surgery may further reduce surgical invasiveness, their application in scirrhous gastric cancer remains challenging owing to marked fibrosis, diffuse gastric wall thickening, and limitations in intraoperative margin assessment. Continued technical refinement and growing institutional experience will be essential to safely expand these approaches.

Taken together, the future role of splenectomy in scirrhous gastric cancer is likely to shift from a routine component of D2 lymphadenectomy to a more selective procedure based on individual tumor characteristics and treatment context. A balanced strategy that integrates advances in systemic therapy, individualized surgical decision‐making, and minimization of surgical morbidity will be essential to optimize outcomes in this challenging disease.

## 9 Conclusions

Scirrhous gastric cancer remains a highly aggressive disease characterized by diffuse infiltration and a strong propensity for peritoneal dissemination, which limits the impact of locoregional control strategies. Although splenic hilar lymph node metastasis occurs with a relatively high frequency, the oncologic benefit of splenectomy remains uncertain, whereas its association with increased postoperative morbidity has been demonstrated. In the modern treatment era, advances in systemic therapy and minimally invasive surgical techniques have created new opportunities to reconsider the role of splenectomy. Spleen‐preserving splenic hilar lymph node dissection may represent a promising strategy to balance adequate nodal clearance with reduced splenectomy‐related morbidity. Its oncologic role in patients with scirrhous gastric cancer remains to be established. Current evidence supports further investigation of the role of splenectomy according to individual clinicopathological characteristics rather than a uniform surgical strategy. Further prospective studies are required to define the optimal role of splenic hilar lymph node dissection in this challenging disease.

## Author Contributions

**Takaki Yoshikawa:** writing – review and editing, supervision. **Tsutomu Hayashi:** conceptualization, methodology, writing – original draft, writing – review and editing, data curation.

## Funding

The authors have nothing to report.

## Conflicts of Interest

The authors declare no conflicts of interest.

## Data Availability

Data sharing not applicable to this article as no datasets were generated or analysed during the current study.
